# A Simplified Score to Quantify Comorbidity in COPD

**DOI:** 10.1371/journal.pone.0114438

**Published:** 2014-12-16

**Authors:** Nirupama Putcha, Milo A. Puhan, M. Bradley Drummond, MeiLan K. Han, Elizabeth A. Regan, Nicola A. Hanania, Carlos H. Martinez, Marilyn Foreman, Surya P. Bhatt, Barry Make, Joe Ramsdell, Dawn L. DeMeo, R. Graham Barr, Stephen I. Rennard, Fernando Martinez, Edwin K. Silverman, James Crapo, Robert A. Wise, Nadia N. Hansel

**Affiliations:** 1 Pulmonary and Critical Care Medicine, Johns Hopkins University School of Medicine, Baltimore, Maryland, United States of America; 2 Epidemiology, Biostatistics & Prevention Institute, University of Zurich, Zurich, Switzerland; 3 Pulmonary & Critical Care Medicine, University of Michigan, Ann Arbor, Michigan, United States of America; 4 Department of Medicine, National Jewish Health, Denver, Colorado, United States of America; 5 Pulmonary, Critical Care and Sleep Medicine, Baylor College of Medicine, Houston, Texas, United States of America; 6 Pulmonary and Critical Care Medicine, Morehouse School of Medicine, Atlanta, Georgia, United States of America; 7 Pulmonary, Allergy and Critical Care Medicine, University of Alabama at Birmingham, Birmingham, Alabama, United States of America; 8 Pulmonary and Critical Care, National Jewish Health, Denver, Colorado, United States of America; 9 Pulmonary and Critical Care Medicine, University of California San Diego, San Diego, California, United States of America; 10 Channing Division of Network Medicine, Brigham and Women's Hospital, Harvard Medical School, Boston, Massachusetts, United States of America; 11 Department of Medicine, Department of Epidemiology, Columbia University Medical Center, New York City, New York, United States of America; 12 Pulmonary and Critical Care Medicine, University of Nebraska Medical Center, Omaha, Nebraska, United States of America; 13 Pulmonary and Critical Care Medicine, New York Presbyterian-Weill Cornell Medical College, New York City, New York, United States of America; Instituto de Higiene e Medicina Tropical, Portugal

## Abstract

**Importance:**

Comorbidities are common in COPD, but quantifying their burden is difficult. Currently there is a COPD-specific comorbidity index to predict mortality and another to predict general quality of life. We sought to develop and validate a COPD-specific comorbidity score that reflects comorbidity burden on patient-centered outcomes.

**Materials and Methods:**

Using the COPDGene study (GOLD II-IV COPD), we developed comorbidity scores to describe patient-centered outcomes employing three techniques: 1) simple count, 2) weighted score, and 3) weighted score based upon statistical selection procedure. We tested associations, area under the Curve (AUC) and calibration statistics to validate scores internally with outcomes of respiratory disease-specific quality of life (St. George's Respiratory Questionnaire, SGRQ), six minute walk distance (6MWD), modified Medical Research Council (mMRC) dyspnea score and exacerbation risk, ultimately choosing one score for external validation in SPIROMICS.

**Results:**

Associations between comorbidities and all outcomes were comparable across the three scores. All scores added predictive ability to models including age, gender, race, current smoking status, pack-years smoked and FEV_1_ (p<0.001 for all comparisons). Area under the curve (AUC) was similar between all three scores across outcomes: SGRQ (range 0·7624–0·7676), MMRC (0·7590–0·7644), 6MWD (0·7531–0·7560) and exacerbation risk (0·6831–0·6919). Because of similar performance, the comorbidity count was used for external validation. In the SPIROMICS cohort, the comorbidity count performed well to predict SGRQ (AUC 0·7891), MMRC (AUC 0·7611), 6MWD (AUC 0·7086), and exacerbation risk (AUC 0·7341).

**Conclusions:**

Quantifying comorbidity provides a more thorough understanding of the risk for patient-centered outcomes in COPD. A comorbidity count performs well to quantify comorbidity in a diverse population with COPD.

## Introduction

Chronic obstructive pulmonary disease (COPD) is a major cause of morbidity and mortality, recently becoming the third leading cause of death in the US. [1] Comorbidities are common in COPD [2] and have been shown to be associated with mortality, [3], [4] poor quality of life [5]–[7] and worse health status. [8]

Disease-specific instruments [9]–[11] for measuring comorbidity burden have more utility for predicting clinical events and mortality than general instruments. [12] The COTE (COPD specific comorbidity test) index was recently developed in a COPD population to use comorbidities for predicting mortality, [3] and another index was recently developed to describe general quality of life in COPD. [13] Currently, there is no externally validated disease-specific comorbidity index in COPD for predicting patient-reported clinical outcomes, particularly relevant given the importance of patient-centered outcomes in chronic disease populations such as COPD.

We hypothesized that the wealth of phenotypic data collected in the COPDGene (Genetic Epidemiology of COPD) [14] and SPIROMICS (Subpopulations and intermediate outcome measures in COPD study) [15] studies would enable development and external validation of a score to describe and quantify the impact of comorbidity burden on respiratory disease-specific quality of life. This score could help clinicians with prognostication and risk stratification of COPD in the clinic as well as serve as a valuable research tool.

## Materials and Methods

### Study population

#### COPDGene

COPDGene is a multicenter observational study of 10,192 current and former smokers with 10 or more pack-years and age 45–80 years with and without COPD. The study protocol and goals have been previously published. [14] Participants were of non-Hispanic White or African-American race. Exclusion criteria included lung disease other than asthma, lung resection or lung volume reduction surgery, pregnancy, cancer being actively treated or suspected lung cancer. We examined data of individuals with GOLD stages II-IV COPD (post-bronchodilator spirometry with FEV_1_/FVC<0.7 and FEV_1_<80% predicted).

#### SPIROMICS

SPIROMICS [15] is a multicenter observational study recruiting smokers (with 20 or more pack-years smoking history) and non-smokers age 40–80 years with and without COPD. We studied individuals recruited into SPIROMICS strata three and four, which included mild through severe COPD (GOLD spirometry class I–IV), based upon the presence of FEV_1_/FVC ratio of <70% on post-bronchodilator spirometry. Exclusion criteria for the study were non-COPD obstructive lung disease, BMI>40 kg/m^2^, history of lung surgery, or intolerance to bronchodilators. Participant selection for both COPDGene and SPIROMICS studies is depicted in S1 Figure.

### Comorbidity assessment

We evaluated fourteen comorbidities, with comorbidity being defined as a condition (disease or risk factor) requiring treatment or management while having the potential of impacting “a person's physical and emotional well-being.” [16] In both cohorts, comorbidities were assessed by self-report of physician diagnosis with the exception of obesity, which was determined using measured height and weight (S1 Table describes definitions of comorbidities). We included some conditions considered to be risk factors for disease such as high cholesterol, obesity and hypertension within our group of comorbidities. We included those conditions which were present in 3% or more of the training cohort, also having some amount of specificity(e.g. including sleep apnea but not cancer because cancer reflects a broad spectrum of diseases with variable prognoses and treatments).

### Outcomes

The primary outcome of interest was respiratory disease-specific quality of life as measured by St. George's Respiratory Questionnaire(SGRQ), where higher scores indicate worse health status. The SGRQ total score has been previously validated [17], with a minimally important difference of four units. [18]

Secondary outcomes included exercise capacity measured using a standardized protocol for six minute walk distance in feet, [19] dyspnea measured with the validated modified Medical Research Council questionnaire (mMRC), [20] and exacerbation risk. In both cohorts, individuals were classified as at risk for exacerbations if they retrospectively reported at least one exacerbation in the past year requiring change in medications, unscheduled visit to the doctor or healthcare facility or hospitalization.

### Statistical analyses

#### Population characteristics

We compared baseline characteristics of individuals with three or more comorbidities to individuals with those having two or fewer comorbidities (given three was the mean number of comorbidities in both cohorts) using t-tests and chi-squared tests to calculate p-values for comparisons.

#### Comorbidity associations and score development using the COPDGene cohort

We tested individual associations of each comorbidity using regression models with the primary outcome of interest, SGRQ, first unadjusted and then adjusted for age, race, gender, FEV_1_ percent predicted, pack-years smoked and current smoking status. We also performed backwards stepwise selection, locking in the above adjusters, while simultaneously including all comorbidities, with criteria for exclusion being a p-value of>0·2. We then developed three candidate comorbidity scores for further comparisons:

1. A sum of number of comorbidities, with number of comorbidities determined by available data on comorbidities in the cohort.
2. A weighted comorbidity score, where each comorbidity received a weight based upon the coefficient from the models of adjusted association (all comorbidities included)
3. A weighted comorbidity score, where only comorbidities left after the stepwise selection procedure were included, and each included comorbidity received a weight based upon the coefficient from the stepwise selection procedure

#### Internal validation

Once all three scores were developed, measures of discrimination and calibration were computed within the same participants. For discrimination, we first fit an “empty” model without comorbidities that included only the covariates noted above (age, race, gender, current smoking status, pack-years smoked and FEV1 % predicted, described in Table 1) and calculated area under the curve (AUC). We then computed AUCs of separate models including each comorbidity score separately along with covariates. We compared the separate models (empty model versus models with each separate score, then comparing each score to the other using the “roccomp” command in Stata [21] to compute p-values for the comparisons). We calculated Hosmer-Lemeshow (H-L) calibration statistics with p-values to assess calibration, in this case a higher p-value indicates better calibration. For validation steps, continuous outcomes were dichotomized at their group mean in order to utilize logistic regression models for calculation of AUCs and H-L statistics. At the end of this step we reviewed the association and validation data for all three scores and chose one score for external validation steps based upon performance.

**Table 1:** Characteristics of participants with COPD based upon number of comorbidities.

	COPDGene			SPIROMICS		
	≤2 comorbidites	>2 comorbidities	p-value	≤2comorbidites	>2 comorbidities	p-value
N	1,733 (47)	1,954 (53)		426 (50)	427 (50)	
Age, years	62 (8.7)	65 (8.1)	<0·001	66 (7·9)	67 (7·6)	0·287
African American, n(%)	457 (26)	379 (19)	<0·001	54 (13)	53 (12)	0·907
Hispanic, n(%)	NA	NA	NA	11 (3)	21 (5)	0·073
Pack-years smoked	50·0 (25·7)	55.6 (28.8)	<0·001	53·1 (25·5)	56·0 (27·6)	0·120
BMI, kg/m^2^	25·7 (5·0)	30·1 (6·5)	<0·001	24·8 (3·8)	30·1 (5·0)	<0·001
Post-BD FEV1 percent predicted	49·9 (19·2)	50·5 (16·8)	0·295	59·4 (23·7)	62·7 (22·1)	0·044
Current smokers, n(%)	851 (49)	649 (33)	<0·001	172 (40)	107 (25)	<0·001
MMRC dyspnea score	1·9 (1·4)	2·3 (1·4)	<0·001	1·1 (1·0)	1·2 (0·9)	0·126
6MWD, meters	374·6 (121·5)	343·2 (118·6)	<0·001	410·0 (107·1)	381·6 (115·9)	<0·001
SGRQ score	37·9 (22·4)	43·6 (21·1)	<0·001	34·4 (19·7)	36·4 (18·3)	0·152
Exacerbation in past yr, n(%)	588 (34)	938 (48)	<0·001	106 (25)	124 (29)	0·214
Oxygen Therapy, n(%)	417 (24.1)	614 (31.4)	<0.001	50 (14.5)	110 (24.6)	<0.001
Use of Inhaled Corticosteroids, n(%)	788 (46.5)	1,100 (57.1)	<0.001	140 (40.6)	196 (43.9)	0.34
Use of Oral Corticosteroids, n(%)	87 (5.2)	123 (6.6)	0.09	10 (2.9)	20 (4.5)	0.24

All values as mean(SD) unless otherwise indicated.

#### External validation using the SPIROMICS cohort

We computed the comorbidity score chosen from above steps in the first 853 COPD subjects recruited as part of the SPIROMICS cohort. High cholesterol, stomach ulcers and peripheral vascular disease were assessed in COPDGene but not in SPIROMICS. Accordingly these conditions were dropped from the score for external validation only. We then computed measures of association (adjusted regression models), discrimination (AUCs) and calibration (H-L statistics) as described in the internal validation steps above, with regards to all described outcomes (SGRQ, 6MWD, mMRC, exacerbation risk). Continuous outcomes were dichotomized at their group mean (for validation cohort) in order to utilize logistic regression models for calculation of AUCs and H-L statistics.

All analyses were performed using Stata 12 software package. [22] The COPDGene and SPIROMICS studies were approved by Institutional Review Boards at each clinical center and all participants provided written informed consent, with centers and approval numbers listed in S1 Table. Both studies are observational studies but have been registered on clinicaltrials.gov: NCT01969344 (SPIROMICS) and NCT00608764 (COPDGene).

## Results

### Cohort characteristics and comorbidity burden

Characteristics of participants in the COPDGene and SPIROMICS study populations were compared based upon presence of two or fewer versus three and more comorbidities (Table 1). Individuals with more comorbidities had higher BMI and had more cumulative smoking history but were less likely to be current smokers. In the COPDGene population, individuals with more comorbidities were older and less likely to be African American. Overall, prevalence of comorbidities was comparable in the two cohorts, with the most prevalent conditions being hypertension (51% and 50% in COPDGene and SPIROMICS, respectively), GERD (30% in both cohorts) and obesity (33% and 32% in COPDGene and SPIROMICS, respectively; S2 Table).

### Association of comorbidities with SGRQ and score development using COPDGene

We computed individual associations between each comorbidity and SGRQ, adjusting for age, race, gender, FEV1 percent predicted, pack-years smoked and current smoking status. All conditions are significantly associated with worse health status (measured by SGRQ total score), with sleep apnea (8·83 points higher on SGRQ scale, 95% CI 7·22–10·43), CHF (6·53 points higher, 95% CI 3·82–9·24) and GERD (6·45 points higher, 95% CI 5·11–7·78) having the strongest negative impact on health status (S3 Table).

We formulated three candidate comorbidity scores as noted above: sum of the number of comorbidities, weighted score, using coefficients from the individual adjusted associations (S2 Table), and weighted score based upon selection procedure. The average values of the scores were 2·9 (SD 2·1, range 0–11) for the comorbidity count, 12·9 (SD 10·0, range 0–54.8) for the weighted score, and 6·2 (SD 5·3, range 0–29.6) for the weighted score from selection, with differences in values reflecting differences in how the scores were constructed. All three scores were significantly associated with higher SGRQ values (p<0·001). For example, an increase in comorbidity count by one point was associated with an increase in SGRQ by 2·31 points (95% CI 2·01–2·60; Table 2).

**Table 2:** Mean, SD and adjusted associations of comorbidity scores and SGRQ value using COPDGene.

	Mean (SD)	Coefficient	95% CI	p-value
Comorbidity count	2·9 (2·1)	2·31	(2·01, 2·60)	<0·001
Weighted comorbidity score	12·9 (10·0)	0·52	(0·45, 0·58)	<0·001
Weighted score from selection	6·2 (5·3)	1·00	(0·89, 1·11)	<0·001

Coefficients adjusted for age, race, FEV1, pack-years smoked, current smoking status and gender.

Equation for weighted comorbidity score: (4·93*coronary heart disease) + (4·69*diabetes) + (6·53*congestive heart failure) + (5·96*stroke) + (5·13*osteoarthritis) + (4·31*osteoporosis) + (3·24*hypertension) + (2·14*high cholesterol) + (6·45*GERD) + (4·94*stomach ulcers) + (5*obesity) + (8·83*sleep apnea) + (2·75*hay fever) + (3·71*peripheral vascular disease).

Equation for weighted score based on backwards selection: (2·16*coronary heart disease) + (1·39*diabetes) + (2·37*congestive heart failure) + (4·71*stroke) + (2·35*osteoarthritis) + (3·29*osteoporosis) + (0·89*hypertension) + (4·13*GERD) + (2·48*stomach ulcers) + (2·69*obesity) + (6·49*sleep apnea) + (1·20*hay fever).

### Internal validation

#### SGRQ

**Figure 1:**
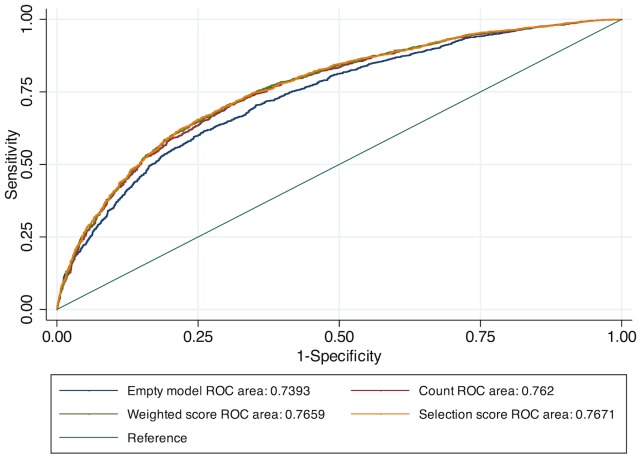
Areas Under the Curve (AUCs) for discrimination of comorbidity scores with regards to primary outcome SGRQ. All three scores compared to “empty” model (including age, gender, race, FEV1, pack-years smoked and current smoking status). ROC for empty model is 0.7393.

AUCs were significantly higher in models including comorbidity scores as compared to the ‘empty’ model (p<0·001 for all comparisons). Discrimination and calibration statistics of the three comorbidity scores were similar and each performed reasonably well to predict SGRQ (for comorbidity count AUC 0·7624, HL statistic 3·61, p-value 0·89; for weighted score AUC 0·7665, HL statistic 5·90, p-value 0·66; for weighted score from selection AUC 0·7676, HL statistic 2·65, p-value 0·95; Fig. 1).

#### Exacerbations, dyspnea, and exercise capacity

The three comorbidity scores were significantly associated with all secondary outcomes (MMRC, 6MWD and exacerbations), with p-values for all comparisons being <0·001 (Table 3). For example, a one point increase in the comorbidity count was associated with a 21% increased risk for an exacerbation (95% CI 1·17, 1·26), 20% increased risk for worse dyspnea score (95% CI 1·17, 1·24), and 10·6 fewer meters walked in six minutes (95% CI −12·1, −8·7). Discrimination was slightly worse for these three outcomes compared to SGRQ, most notably for exacerbations in which AUC ranged from 0·6831–0·6919. Calibration was adequate except in the case of the weighted comorbidity score with regards to the outcome of exacerbations. Sensitivity analysis was performed for the measures of association, calibration and discrimination after excluding those reporting current asthma from the population with COPD and the results were minimally changed (data not shown).

**Table 3:** Discrimination measures (AUC) and calibration measures (Hosmer-Lemeshow calibration statistics) for comorbidity scores with regards to exacerbations, MMRC, and 6MWD, using COPDGene.

	Association with outcome		AUC	HL statistic	p-value for HL statistic
**Exacerbations**	**OR**	**95% CI**			
Comorbidity count	1·21	(1·17, 1·26)	0·6831	13·24	0·1040
Weighted comorbidity score	1·044	(1·037, 1·052)	0·6880	20·35	0·0091
Weighted score from selection	1·09	(1·07, 1·10)	0·6919	11·02	0·2007
**MMRC**	**OR**	**95% CI**			
Comorbidity count	1·20	(1·17, 1·24)	0·7590	9·42	0·3078
Weighted comorbidity score	1·042	(1·037, 1·049)	0·7632	12·04	0·1494
Weighted score from selection	1·08	(1·07, 1·09)	0·7644	15·44	0·0511
**6MWD**	**β**	**95% CI**			
Comorbidity count	−34·0	(−39·6, −28·5)	0·7531	10·60	0·2253
Weighted comorbidity score	−7·7	(−8·9, −6·6)	0·7560	10·75	0·2161
Weighted score from selection	−4·3	(−5·0, −3·7)	0·7551	5·60	0·6919

Above models also include terms for age, gender, race, baseline FEV1, pack-years smoked and current smoking status. Every score above added to “empty” model, with addition of score improving AUC significantly (p<0·001 for all comparisons) with ROCs for empty models as follows: exacerbations 0.6542, MMRC 0.7423, 6MWD 0.7392.For associations with outcome, OR for exacerbations represents risk for exacerbation conferred by one point increase in comorbidity score, OR for MMRC represents risk for worse dyspnea score conferred by one point increase in comorbidity score, and β for 6MWD represents decrement in exercise capacity (in meters walked) conferred by one point increase in comorbidity score. All ROCs estimated using logistic regression with outcomes of SGRQ (group mean 40.90, SD 21.89), MMRC (group mean 2.14, SD 1.41) and 6MWD (group mean 358.1, SD 121.0)dichotomized at group mean.

### External validation using the SPIROMICS cohort

Based upon the above results, we performed external validation of the comorbidity count. Mean comorbidity count in the SPIROMICS cohort was 2·7 (SD 1·8), with range from 0–11 reflecting the three comorbidities not assessed in SPIROMICS compared to COPDGene. The association of the comorbidity count was strongly and significantly associated with all four outcomes (Table 4). A one point increase in comorbidity count was associated with a two point increase in SGRQ (95% CI 1·36, 2·63), a 16% increase in exacerbation risk (95% CI 1·04, 1·28), 23% risk for worse dyspnea (95% CI 1·13, 1·34), and 13·1 fewer meters walked in six minutes (95% CI −17·3, −8·9). Calibration of the score was appropriate for all outcomes (HL statistic p-value range 0·3849–0·9164) and the comorbidity count performed well to discriminate differences in all four outcomes, most remarkably for SGRQ, where AUC was 0·7891. We performed measures of discrimination, calibration and association with the weighted comorbidity score and weighted score from selection to confirm that the comorbidity count was a reasonable choice and found similar results as in the COPDGene cohort (S4 Table). We performed sensitivity analyses dichotomizing the continuous outcomes at the group mean for the COPDGene cohort for these steps and the results of our analyses were minimally changed (data not shown).

**Table 4:** Discrimination measures (AUC) and calibration measures (Hosmer-Lemeshow calibration statistics) for comorbidity count with regards to outcomes of exacerbations, MMRC, and 6MWD, in the SPIROMICS participants.

	Association with outcome		AUC	HL statistic	p-value for HL statistic
**SGRQ**	**β**	**95% CI**			
Comorbidity count	2·00	(1·36, 2·63)	0·7891	3·50	0·8991
**Exacerbations**	**OR**	**95% CI**			
Comorbidity count	1·16	(1·04, 1·28)	0·7341	4·11	0·8472
**MMRC**	**OR**	**95% CI**			
Comorbidity count	1·23	(1·13, 1·34)	0·7611	8·51	0·3849
**6MWD**	**β**	**95% CI**			
Comorbidity count	−13·1	(−17·3, −8·9)	0·7086	3·27	0·9164

Above models also include terms for age, gender, race, baseline FEV1, pack-years smoked and current smoking status. Every score above added to “empty” model, with addition of score improving AUC significantly (p<0·001 for all comparisons). The AUCs for the empty models are as follows: SGRQ 0.7741, Exacerbations 0.7223, MMRC 0.7499, 6MWD 0.6970. For associations with outcome, OR for exacerbations represents risk for exacerbation conferred by one point increase in comorbidity score, OR for MMRC represents risk for worse dyspnea score conferred by one point increase in comorbidity score, and β's for SGRQ and 6MWD represent decrement in health status and exercise capacity conferred by one point increase in comorbidity score. All ROCs estimated using logistic regression with outcomes of SGRQ (group mean 35.4, SD 18.9), MMRC (group mean 1.18, SD 0.99) and 6MWD (group mean 395.5, SD 112.5) dichotomized at group mean.

## Discussion

Using COPD cohorts of two large multicenter studies, we have developed a COPD-specific comorbidity score which adds significant predictive ability to models predicting clinical outcomes in COPD. A simple count of number of comorbidities is a simplified way to quantify comorbidity in COPD and can be easily applied to other cohorts of COPD with different characteristics and spectrum of disease.

Accounting for comorbidity adds value to other known determinants of health status in COPD. After adding the comorbidity count to a model already including age, race, gender, lung function, current smoking status and cumulative smoking history, the model discrimination for SGRQ improved with p-value of <0.01 for statistical comparison with base model. The comorbidity score improved the prediction of health status, and three other outcomes of importance to patients with COPD: dyspnea, exercise capacity and exacerbation risk. The addition of comorbidities leads to changes in AUC values that may appear to be small. However, the AUC is rather insensitive to model improvements and additional predictors need to be strongly associated to change the AUC compared to a base model. Previous studies have found that even small increases in AUC of 0.02 correlate with clinically relevant changes in classification and diagnosis of individuals. [23] Indeed, the regression models (Table 2 and S4 Table) suggest that comorbidities independently and strongly impact quality of life, dyspnea, exercise capacity and exacerbation risk, when added to smoking, gender, lung function, age and race. The fact that prediction is improved with the addition of comorbidity is important and informative in this setting. Overall, it is the combination of the findings of the strong associations in regression models with the findings of the prediction steps of discrimination and calibration that underscores the importance of comorbidity burden on patient-centered outcomes in COPD.

Further, clinical studies of COPD are increasingly focused upon understanding risk for clinical outcomes. Because individuals with COPD have a significant burden of comorbidity [2], [3], [8] which appears to contribute to such poor clinical outcomes, quantifying comorbidity burden is of importance in epidemiologic studies and clinical trials. However, contribution of comorbidities to these outcomes, including outcomes such as dyspnea and quality of life that are not specific to COPD, may differ based upon the underlying chronic disease status. For example, obesity may impact individuals with COPD by causing more airways inflammation and change in lung volumes, whereas in a population with cardiovascular disease obesity may have more impacts through metabolic syndrome leading to endothelial dysfunction. Since it is likely that comorbidities act through different mechanisms in different chronic diseases, there is value in the development of a comorbidity score that is relevant to the specific population of individuals with COPD. Validation of comorbidity score was less significant for the predication of exacerbations because comorbidities likely have more impact on the general markers of health such as dyspnea and quality of life, whereas exacerbations are a disease-specific process related to COPD.

The burden of comorbidities can be established by a simple comorbidity count, which requires no equation or complicated mathematical algorithm. As a result, this kind of score is easy to apply in the clinical or research setting. Other general and specific comorbidity indices require calculations using weights. [3], [12] With internal and external validation, important steps in proving the applicability and reliability of prediction models, we have shown that a simple count of comorbidities can perform just as well as a weighted score or score based upon complicated statistical selection procedures.

Finally, we have shown that the comorbidity count not only predicts important clinical outcomes in the COPDGene study population, when applied to a different study population its predictive capability was equally strong. The SPIROMICS study population used in this analysis differs slightly from COPDGene in that participants are slightly older, with slightly better lung function and fewer current smokers. Given that both studies include individuals from a broad range of COPD severity with large percentage of African Americans and females, this scoring system is broadly applicable in the real world setting across a range of COPD severity, racial makeup, and gender. All of these characteristics demonstrate that this comorbidity count is generalizable. Notably, we tested our comorbidity index on the population of at-risk smokers without COPD who are enrolled in both the COPDGene and SPIROMICS cohorts and found that there may be utility of this index in describing comorbidity in the at-risk population of smokers without spirometric evidence of COPD, though our findings are predictably less robust in at risk populations than in the population with COPD (S5 Table and S6 Table).

Our study has limitations. First, we relied on self-report to define most comorbidities for this study. For large scale studies, assessment of comorbidity using medical records is cumbersome and costly. The validity of self-report does differ based upon the characteristics of the population studied and the comorbidity considered [24]–[29] and should be considered when interpreting the results of this study. In addition, we are limited by the lack of data on severity of comorbidities, and have assessed them simply as present or absent. More quantitative information on comorbidity severity including data on how comorbidities are treated could have the potential to enhance the utility or performance of such a score. Also, we did not include depression and anxiety as comorbidities since these data were not available. The other existing comorbidity index to predict generic quality of life, the COMCOLD index, [13] showed that depression and anxiety are among the strongest predictors of health-related quality of life. Additionally, if data on severity and treatment of comorbidities were available its possible that a weighted score would have been more useful in predicting outcomes. We also did not have data on cognitive impairment, an increasingly recognized comorbidity of the COPD population. [30] Further, though mental illness such as depression and anxiety were assessed in SPIROMICS, lack of data on this comorbidity in COPDGene limited our ability to include it in our index. However, when we incorporated these conditions for the external validation steps (requiring recalibration of the index specific to the SPIROMICS population), the comorbidity count continued to be comparable to the more complicated comorbidity index measures (data not shown). Further, lack of data on mental illness, as well as insufficient data on malignancies in COPDGene limited our ability to compare the performance of the comorbidity count to indices of comorbidity in COPD such as the COTE index. Finally, we have constructed and validated this score in a broad range of COPD patients. However, given the increasingly recognized heterogeneity of COPD and the likelihood that some comorbidities are more common in subsets of COPD patients [31], it is possible that our score may perform slightly better or worse in any one subset of COPD patients. In addition, because of the variability and inconsistency in the comorbidities assessed in these two study populations, it was impossible to formulate and validate an index with specific cutoffs for the prediction of clinical endpoints in COPD. Moving forward the next step would be to develop and externally validate such an index in study populations with consistent data on a broad range of comorbidities, testing cutoffs for the prediction of relevant clinical outcomes such as quality of life and dyspnea.

Our study did have several strengths. First, these results derive from two large, multicenter, well-characterized and fairly representative populations of individuals with COPD, making our results generalizable. Second, our finding that comorbidity in COPD can be quantified with such ease has the potential to improve and simplify the measurement of comorbidity in future studies and thus can have far-reaching effects. Next, we have focused upon clinically-relevant outcomes, which are important to patients with chronic disease such as COPD. Finally, by validating our score internally and externally we have undertaken an important step not demonstrated before in the COPD comorbidity literature.

## Conclusions

We have shown that a comorbidity count in individuals with COPD is not only simplified, but when added to known risk factors is also predictive of important outcomes such as health status, dyspnea, exercise capacity and exacerbation risk. Moving forward, validation of a similar index in populations with a broad range of COPD severity could add generalizability and validity to our preliminary findings that a comorbidity count is a simple and relevant way to quantify comorbidity burden in COPD. Ultimately, such an index can have broad applicability to help quantify comorbidity burden in COPD clinical research. More importantly, such a score can assist clinicians to identify populations with COPD at risk for poor health related outcomes, so that interventions that improve quality of life, dyspnea and exercise capacity, such as rehabilitation, can be considered earlier.

## Supporting Information

S1 Figure **Participant selection for SPIROMICS and COPDGene studies.**
(DOCX)

S1 Table **Clinical Center IRB Approvals.**
(DOCX)

S2 Table **Prevalence and definition of comorbidities in COPD participants of COPDGene and SPIROMICS cohorts.**
(DOCX)

S3 Table **Adjusted associations between individual comorbidities and SGRQ total score, COPDGene.**
(DOCX)

S4 Table **Discrimination measures (AUC) and calibration measures (Hosmer-Lemeshow calibration statistics) for comorbidity scores with regards to outcomes of exacerbations, MMRC, and 6MWD, in the SPIROMICS participants.**
(DOCX)

S5 Table **Discrimination measures (AUC) and calibration measures (Hosmer-Lemeshow calibration statistics) for comorbidity scores with regards to exacerbations, MMRC, and 6MWD, using COPDGene former and current smoking controls (current and former smokers without COPD).**
(DOCX)

S6 Table **Discrimination measures (AUC) and calibration measures (Hosmer-Lemeshow calibration statistics) for comorbidity count with regards to outcomes of exacerbations, MMRC, and 6MWD, in the former and current smoking control SPIROMICS participants (current and former smokers without COPD).**
(DOCX)
