# Supplementary figures and images for: A Simplified Score to Quantify Comorbidity in COPD
Source: PLoS One. 2014 Dec 16;9(12):e114438. doi: 10.1371/journal.pone.0114438 (PMC4267736; doi:10.1371/journal.pone.0114438)

Figure S1: Participant selection for SPIROMICS and COPDGene studies.


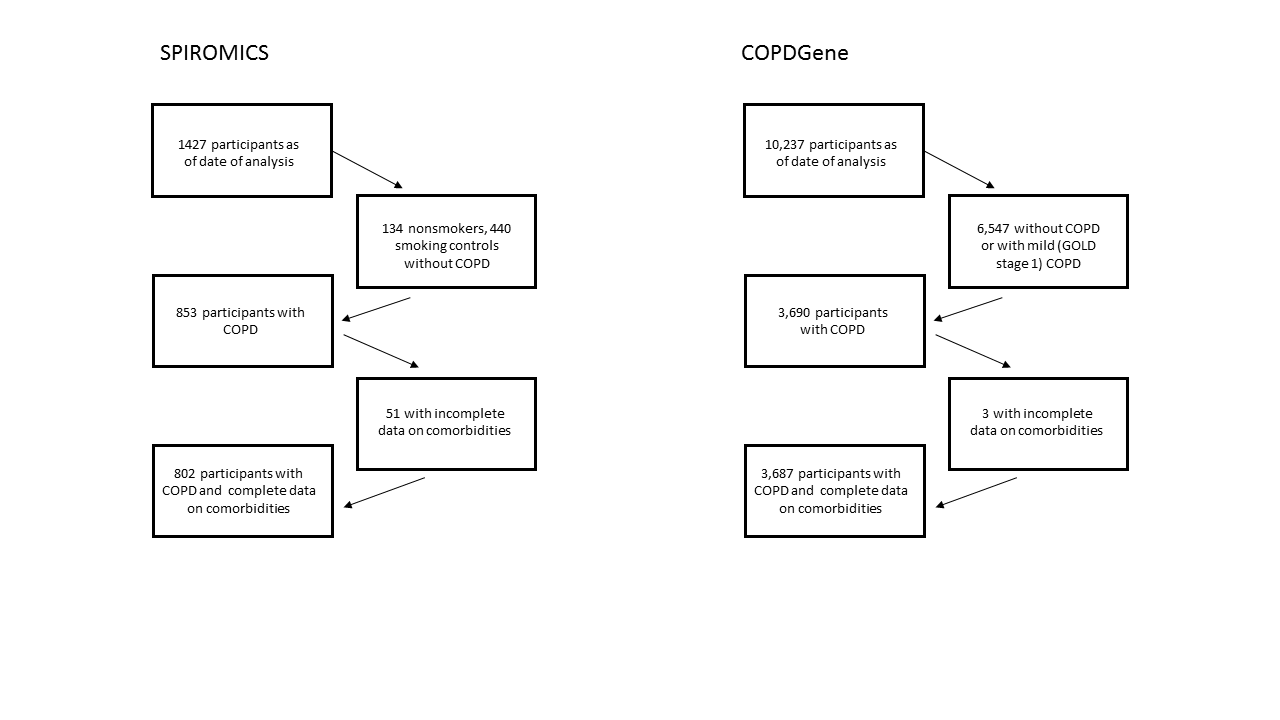

Supplement: S1 Figure — Participant selection for SPIROMICS and COPDGene studies. (DOCX) [file pone.0114438.s001.docx]
