# Supplementary material for: A Simplified Score to Quantify Comorbidity in COPD
Source: PLoS One. 2014 Dec 16;9(12):e114438. doi: 10.1371/journal.pone.0114438 (PMC4267736; doi:10.1371/journal.pone.0114438)
Supplement: S1 Table — Clinical Center IRB Approvals. (DOCX) [file pone.0114438.s002.docx]

| Table S1: Clinical Center IRB Approvals | | |
| --- | --- | --- |
| **Clinical Center** | **Institution Title** | **Protocol Number** |
| National Jewish Health | National Jewish IRB | HS-1883a |
| Brigham and Women’s Hospital | Partners Human Research Committee | 2007-P-000554/2; BWH |
| Baylor College of Medicine | Institutional Review Board for Baylor  College of Medicine and Affiliated Hospitals | H-22209 |
| Michael E. DeBakey VAMC | Institutional Review Board for Baylor College of Medicine and Affiliated Hospitals | H-22202 |
| Columbia University Medical Center | Columbia University Medical Center IRB | IRB-AAAC9324 |
| Duke University Medical Center | The Duke University Health System Institutional Review Board for Clinical Investigations (DUHS IRB) | Pro00004464 |
| Johns Hopkins University | Johns Hopkins Medicine Institutional Review Boards (JHM IRB) | NA_00011524 |
| Los Angeles Biomedical Research Institute | The John F. Wolf, MD Human Subjects Committee of Harbor-UCLA Medical Center | 12756-01 |
| Morehouse School of Medicine | Morehouse School of Medicine Institutional Review Board | 07-1029 |
| Temple University | Temple University Office for Human Subjects Protections Institutional Review Board | 11369 |
| University of Alabama at Birmingham | The University of Alabama at Birmingham Institutional Review Board for Human Use | FO70712014 |
| University of California, San Diego | University of California, San Diego Human Research Protections Program | 070876 |
| University of Iowa | The University of Iowa Human Subjects Office | 200710717 |
| Ann Arbor VA | VA Ann Arbor Healthcare System IRB | PCC 2008-110732 |
| University of Minnesota | University of Minnesota Research Subjects’ Protection Programs (RSPP) | 0801M24949 |
| University of Pittsburgh | University of Pittsburgh Institutional Review Board | PRO07120059 |
| University of Texas Health Sciences Center at San Antonio | UT Health Science Center San Antonio Institutional Review Board | HSC20070644H |
| Health Partners Research Foundation | Health Partners Research Foundation Institutional Review Board | 07-127 |
| University of Michigan | Medical School Institutional Review Board (IRBMED) | HUM00014973 |
| Minneapolis VA Medical Center | Minneapolis VAMC IRB | 4128-A |
| Fallon Clinic | Institutional Review Board/Research Review Committee Saint Vincent Hospital – Fallon Clinic – Fallon Community Health Plan | 1143 |
