# Supplementary material for: A Simplified Score to Quantify Comorbidity in COPD
Source: PLoS One. 2014 Dec 16;9(12):e114438. doi: 10.1371/journal.pone.0114438 (PMC4267736; doi:10.1371/journal.pone.0114438)
Supplement: S2 Table — Prevalence and definition of comorbidities in COPD participants of COPDGene and SPIROMICS cohorts. (DOCX) [file pone.0114438.s003.docx]

| Table S2: Prevalence and definition of comorbidities in COPD participants of COPDGene and SPIROMICS cohorts. | | | |
| --- | --- | --- | --- |
| Comorbidity | Definition | Prevalence in COPDGene | Prevalence in SPIROMICS |
| N |  | 3,690 | 853 |
| Coronary Heart disease (CHD) | Report of angina, coronary artery disease, heart attack, bypass surgery or angioplasty procedure. | 608 (16) | 177 (21) |
| Diabetes | Report of diabetes. | 482 (13) | 114 (13) |
| Congestive heart failure (CHF) | Report of congestive heart failure | 198 (5) | 21 (2) |
| Stroke | Report of stroke or TIA. | 220 (6) | 35 (4) |
| Osteoarthritis | Report of osteoarthritis. | 764 (21) | 246 (29) |
| Osteoporosis | Report of osteoporosis or report of compression fractures in back | 640 (17) | 107 (13) |
| Hypertension | Report of high blood pressure. | 1868 (51) | 428 (50) |
| High cholesterol | Report of high cholesterol | 1529 (41) | NA |
| Gastroesophageal reflux disease (GERD) | Report of gastroesophageal reflux disease. | 1116 (30) | 254 (30) |
| Stomach ulcers* | Report of stomach ulcers. | 372 (11) | NA |
| Obesity | Measured BMI of greater than or equal to 30. | 1227 (33) | 270 (32) |
| Sleep apnea | Report of sleep apnea (with or without doctor diagnosis) or treatment of sleep apnea. | 631 (17) | 139 (16) |
| Hay fever | Report of hay fever (with or without doctor diagnosis) or treatment of hay fever. | 900 (24) | 236 (28) |
| Peripheral Vascular Disease (PVD) | Report of peripheral vascular disease. | 126 (3) | NA |
| All prevalence values displayed as n (%). Shaded cells indicate lack of data on specified comorbidity in SPIROMICS cohort. The questionnaires from which the above data is collected for COPDGene can be found at <http://www.copdgene.org/sites/default/files/COPDGene_Medical_History_Public.pdf>. The corresponding questionnaires for SPIROMICS can be found at <http://www.cscc.unc.edu/spir/public/UNLICOMMBMHBaselineMedicalHistoryForm08252011.pdf>.  *Because the SPIROMICS study asks participants about “ulcers” and the COPDGene study asks specifically about “stomach ulcers,” we did not include this comorbidity from SPIROMICS due to the concern for misclassification. | | | |
