# Supplementary material for: A Simplified Score to Quantify Comorbidity in COPD
Source: PLoS One. 2014 Dec 16;9(12):e114438. doi: 10.1371/journal.pone.0114438 (PMC4267736; doi:10.1371/journal.pone.0114438)
Supplement: S3 Table — Adjusted associations between individual comorbidities and SGRQ total score, COPDGene. (DOCX) [file pone.0114438.s004.docx]

| Table S3: Adjusted associations between individual comorbidities and SGRQ total score, COPDGene. | | | |
| --- | --- | --- | --- |
| Comorbidity | Difference in SGRQ score (Coefficient) | 95% Confidence Interval | p-value |
| CHD | 4·93 | (3·26, 6·61) | <0·001 |
| Diabetes | 4·69 | (2·87, 6·50) | <0·001 |
| CHF | 6·53 | (3·82, 9·24) | <0·001 |
| Stroke | 5·96 | (3·39, 8·54) | <0·001 |
| Osteoarthritis | 5·13 | (3·61, 6·66) | <0·001 |
| Osteoporosis | 4·31 | (2·65, 5·97) | <0·001 |
| Hypertension | 3·24 | (2·00, 4·48) | <0·001 |
| High Cholesterol | 2·14 | (0·87, 3·41) | 0·001 |
| GERD | 6·45 | (5·11, 7·78) | <0·001 |
| Stomach ulcers | 4·94 | (2·92, 6·95) | <0·001 |
| Obesity | 5·00 | (3·70, 6·29) | <0·001 |
| Sleep Apnea | 8·83 | (7·22, 10·43) | <0·001 |
| Hay fever | 2·75 | (1·33, 4·17) | <0·001 |
| PVD | 3·71 | (0·34, 7·08) | 0·031 |
| Analyses adjusted for age, race, FEV1, pack-years smoked, current smoking status, gender. | | | |
