# Supplementary material for: A Simplified Score to Quantify Comorbidity in COPD
Source: PLoS One. 2014 Dec 16;9(12):e114438. doi: 10.1371/journal.pone.0114438 (PMC4267736; doi:10.1371/journal.pone.0114438)
Supplement: S4 Table — Discrimination measures (AUC) and calibration measures (Hosmer-Lemeshow calibration statistics) for comorbidity scores with regards to outcomes of exacerbations, MMRC, and 6MWD, in the SPIROMICS participants. (DOCX) [file pone.0114438.s005.docx]

| Table S4: Discrimination measures (AUC) and calibration measures (Hosmer-Lemeshow calibration statistics) for comorbidity scores with regards to outcomes of exacerbations, MMRC, and 6MWD, in the SPIROMICS participants. | | | | | |
| --- | --- | --- | --- | --- | --- |
|  | Association with outcome | | AUC | HL statistic | p-value for HL statistic |
| **SGRQ** | **β** | **95% CI** |  | | |
| Comorbidity count | 2·00 | (1·36, 2·63) | 0·7891 | 3·50 | 0·8991 |
| Weighted comorbidity score | 0.44 | (0.31, 0.57) | 0.7878 | 5.75 | 0.6755 |
| Weighted score from selection | 0.75 | (0.51, 0.98) | 0.7861 | 6.79 | 0.5594 |
| **Exacerbations** | **OR** | **95% CI** |  | | |
| Comorbidity count | 1·16 | (1·04, 1·28) | 0·7341 | 4·11 | 0·8472 |
| Weighted comorbidity score | 1.03 | (1.01, 1.06) | 0.7321 | 10.36 | 0.2405 |
| Weighted score from selection | 1.06 | (1.02, 1.10) | 0.7339 | 8.49 | 0.3875 |
| **MMRC** | **OR** | **95% CI** |  | | |
| Comorbidity count | 1·23 | (1·13, 1·34) | 0·7611 | 8·51 | 0·3849 |
| Weighted comorbidity score | 1.04 | (1.03, 1.06) | 0.7587 | 11.76 | 0.1624 |
| Weighted score from selection | 1.07 | (1.04, 1.11) | 0.7595 | 13.70 | 0.0900 |
| **6MWD** | **β** | **95% CI** |  | | |
| Comorbidity count | -13·1 | (-17·3, -8·9) | 0·7086 | 3·27 | 0·9164 |
| Weighted comorbidity score | -2.9 | (-3.8, -2.0) | 0.7188 | 3.26 | 0.9172 |
| Weighted score from selection | -5.1 | (-6.6, -3.5) | 0.7168 | 5.57 | 0.6949 |
| Above models also include terms for age, gender, race, baseline FEV1, pack-years smoked and current smoking status. For associations with outcome, OR for exacerbations represents risk for exacerbation conferred by one point increase in comorbidity score, OR for MMRC represents risk for worse dyspnea score conferred by one point increase in comorbidity score, and β’s for SGRQ and 6MWD represent decrement in health status and exercise capacity conferred by one point increase in comorbidity score. | | | | | |
