# Supplementary material for: A Simplified Score to Quantify Comorbidity in COPD
Source: PLoS One. 2014 Dec 16;9(12):e114438. doi: 10.1371/journal.pone.0114438 (PMC4267736; doi:10.1371/journal.pone.0114438)
Supplement: S5 Table — Discrimination measures (AUC) and calibration measures (Hosmer-Lemeshow calibration statistics) for comorbidity scores with regards to exacerbations, MMRC, and 6MWD, using COPDGene former and current smoking controls (current and former smokers without COPD). (DOCX) [file pone.0114438.s006.docx]

| Table S5: Discrimination measures (AUC) and calibration measures (Hosmer-Lemeshow calibration statistics) for comorbidity scores using COPDGene former and current smoking controls without COPD. | | | | | |
| --- | --- | --- | --- | --- | --- |
|  | Association with outcome | | AUC | HL statistic | p-value for HL statistic |
| **SGRQ score** | **β** | **95% CI** |  |  |  |
| Comorbidity count | 2.94 | (2.67, 3.21) | 0.7460 | 10.22 | 0.2497 |
| **Exacerbations** | **OR** | **95% CI** |  |  |  |
| Comorbidity count | 1·29 | (1·22, 1·36) | 0·6936 | 4.59 | 0·8001 |
| **MMRC** | **OR** | **95% CI** |  | | |
| Comorbidity count | 1·37 | (1·32, 1·42) | 0·7365 | 12.89 | 0·1159 |
| **6MWD** | **β** | **95% CI** |  |  |  |
| Comorbidity count | -36·7 | (-42·0, -31·3) | 0·7127 | 11.23 | 0·1890 |
| Above models also include terms for age, gender, race, baseline FEV1, pack-years smoked and current smoking status. Every score above added to “empty” model, with addition of score improving AUC significantly (p<0·001 for all comparisons) with ROCs for empty models as follows: SGRQ 0.6971, MMRC 0.6870, exacerbations 0.6507, 6MWD 0.696. For associations with outcome, OR for exacerbations represents risk for exacerbation conferred by one point increase in comorbidity score, OR for MMRC represents risk for worse dyspnea score conferred by one point increase in comorbidity score, and β for 6MWD represents decrement in exercise capacity (in meters walked) conferred by one point increase in comorbidity score. All ROCs estimated using logistic regression with outcomes of SGRQ, MMRC and 6MWD dichotomized at group mean. | | | | | |
