# Supplementary material for: A Simplified Score to Quantify Comorbidity in COPD
Source: PLoS One. 2014 Dec 16;9(12):e114438. doi: 10.1371/journal.pone.0114438 (PMC4267736; doi:10.1371/journal.pone.0114438)
Supplement: S6 Table — Discrimination measures (AUC) and calibration measures (Hosmer-Lemeshow calibration statistics) for comorbidity count with regards to outcomes of exacerbations, MMRC, and 6MWD, in the former and current smoking control SPIROMICS participants (current and former smokers without COPD). (DOCX) [file pone.0114438.s007.docx]

| Table S6: Discrimination measures (AUC) and calibration measures (Hosmer-Lemeshow calibration statistics) for comorbidity count in the former and current smoking control SPIROMICS participants. | | | | | |
| --- | --- | --- | --- | --- | --- |
|  | Association with outcome | | AUC | HL statistic | p-value for HL statistic |
| **SGRQ** | **β** | **95% CI** |  | | |
| Comorbidity count | 3·01 | (2·14, 3.88) | 0·7392 | 11.73 | 0·1635 |
| **Exacerbations** | **OR** | **95% CI** |  | | |
| Comorbidity count | 1·27 | (1·08, 1·49) | 0·7231 | 4·81 | 0·7785 |
| **MMRC** | **OR** | **95% CI** |  | | |
| Comorbidity count | 1·36 | (1·21, 1·52) | 0·7268 | 7.09 | 0·5273 |
| **6MWD** | **β** | **95% CI** |  | | |
| Comorbidity count | -8·2 | (-12·8, -3·6) | 0·6662 | 14.83 | 0·0624 |
| Above models also include terms for age, gender, race, baseline FEV1, pack-years smoked and current smoking status. The AUCs for the empty models with p-value for change after adding comorbidity are as follows: SGRQ 0.6974 (p=0.01), MMRC 0.6814 (p=0.01), 6MWD 0.6332 (p=0.06), exacerbations 0.7030 (p=0.23). For associations with outcome, OR for exacerbations represents risk for exacerbation conferred by one point increase in comorbidity score, OR for MMRC represents risk for worse dyspnea score conferred by one point increase in comorbidity score, and β’s for SGRQ and 6MWD represent decrement in health status and exercise capacity conferred by one point increase in comorbidity score. | | | | | |
